# Impact of COVID-19 Pandemic on Physician-Scientist Trainees to Faculty One Year into the Pandemic

**DOI:** 10.21203/rs.3.rs-3478814/v1

**Published:** 2023-11-08

**Authors:** Aleksandar Obradovic, Omar Toubat, Nathan W. Chen, Aisha Siebert, Carey Jansen, Briana Christophers, Etienne Leveille, Evan Noch, Jennifer M. Kwan

**Affiliations:** Columbia University; O.T, Hospital of the University of Pennsylvania; Yale University; A.S, Northwestern University; Emory University School of Medicine; Weill Cornell Medicine; Yale School of Medicine; Weill Cornell Medicine; Yale School of Medicine

**Keywords:** physician-scientist, COVID-19, medical education, biomedical research

## Abstract

**Purpose:**

Physician-scientists play a crucial role in advancing biomedical sciences. Proportionally fewer physicians are actively engaged in scientific pursuits, attributed to attrition in the training and retention pipeline. This national study evaluated the ongoing and longer-term impact of the COVID-19 pandemic on research productivity for physician-scientists at all levels of training.

**Methods:**

A survey of medical students, graduate students, and residents/fellows/junior faculty (RFJF) was conducted from April to August 2021 to assess the impact of COVID-19 on individual stress, productivity, and optimism. Multivariate regression analyses were performed to identify associated variables and unsupervised variable clustering techniques were employed to identify highly correlated responses.

**Results:**

A total 677 respondents completed the survey, representing different stages of physician-scientist training. Respondents report high levels of stress (medical students: 85%, graduate students: 63%, RFJF: 85%) attributed to impaired productivity concerns, concern about health of family and friends, impact on personal health and impairment in training or career development. Many cited impaired productivity (medical students: 65% graduate students: 79%, RFJF: 78%) associated with pandemic impacts on training, labs closures and loss of facility/resource access, and social isolation. Optimism levels were low (medical students: 37%, graduate students: 38% and RFJF: 39%) with females less likely to be optimistic and more likely to report concerns of long-term effects of COVID-19. Optimism about the future was correlated with not worrying about the long-term effects of COVID-19. Since the COVID-19 pandemic, all respondents reported increased prioritization of time with family/friends (67%) and personal health (62%) over career (25%) and research (24%).

**Conclusions:**

This national survey highlights the significant and protracted impact of the COVID-19 pandemic on stress levels, productivity, and optimism among physician-scientists and trainees. These findings underscore the urgent need for tailored support, including mental health, academic, and career development assistance for this biomedical workforce.

## Introduction

Despite the unique role of physician-scientists in advancing discovery and application of biomedical science to human health, several previous reports suggest that the current number of physician-scientist trainees may not be sufficient to meet expected workforce needs.^1,2^. The physician-scientist training pipeline is long and includes many unique challenges, including financial stressors caused by increasingly delayed matriculation into the workforce, pressure to balance both clinical and scientific productivity, and persistent competition for research funding. In addition to these existing stressors, the presence of additional factors related to COVID-19 may negatively add to the strain experienced by trainees and early career physician-scientists. Given the multiple critical junctures in the physician-scientist training pipeline, it is important to study the effect of these disruptive events and any potential impact they may have contribute on attrition in this already vulnerable group.

We previously reported the results of a national survey examining the effect of COVID-19 on the personal life, career, stress, and productivity amongst physician-scientists at different stages of the training and early-career pipeline (medical student, graduate student, resident, fellow, and early faculty).^3^ Our prior work found that all respondent groups reported high levels of stress, social isolation, and negative impact on productivity resulting from the pandemic. These adverse experiences were disproportionately more likely to be described by women, individuals from disadvantaged backgrounds, and groups traditionally underrepresented in medicine (URM) including Black or African Americans, Hispanic or Latinos, American Indians or Alaskan Natives, Native Hawaiians, and other Pacific Islanders. In addition, some negative consequences of the pandemic, such as social isolation and financial difficulties, were found to be particularly prevalent and disruptive.

Our prior survey data were collected between April and June 2020^3^, capturing immediate and early consequences of the COVID-19 pandemic on trainees and early-career physician-scientists. While our prior work was important for characterizing the early impact of the pandemic, the longer-term consequences of the pandemic and subsequent national response are not known. Understanding the persistent impact of the pandemic on physician-scientist trainees is crucial in order to better support this workforce and potentially mitigate further attrition in this already “leaky” pipeline. Here, we present the results of a national follow-up survey that evaluates the ongoing and longer-term consequences of the COVID-19 pandemic on the personal and professional lives of physician-scientists at all levels of training.

## Methods

### Survey Design and Recruitment

The survey tool was designed with feedback from mental health researchers and academic faculty with expertise in training physician–scientists. Three versions of the survey were tailored to physician-scientist training level for medical students, graduate students, and residents/fellows and junior faculty (RFJF). This study was reviewed and approved as exempt by the Weill Cornell Medicine Institutional Review Board.

From April 2021 to August 2021, the survey was distributed to 120 U.S. institutions with MD–PhD programs by the chairs of the Association of American Medical Colleges’ Group on Graduate Research, Education and Training and by institutional representatives of the American Physician Scientists Association. The survey was administered via SurveyMonkey (Momentive, San Mateo, California).

### Defining Response Variables

For each group of respondents (medical students, graduate students, and RFJF), three outcome measures were defined as an aggregate of response to particular survey questions. Stress outcome was defined as positive if a respondent indicated “Agree” or “Strongly Agree” to any of the following statements:

1. “The COVID-19 pandemic has caused me sleep problems, decreased energy, changes in appetite, difficulty concentrating, and/or restlessness”
2. “The COVID-19 pandemic has caused me a significant amount of stress, anxiety, hopelessness, and/or depression”
3. “Uncertainty of not being able to finish my research or to graduate is a great source of stress”

Productivity outcome was defined as positive if a respondent indicated “Agree” or “Strongly Agree” to any of the following statements:

1. “My research productivity/medical training will be negatively impacted in the long term”
2. “My research productivity/medical training will be negatively impacted in the short term”

Optimism outcome was defined as positive if a respondent indicated “Agree” or “Strongly Agree” to the following statement:

1. “I am optimistic about the future given the trajectory of the COVID-19 pandemic”

### Multivariate Regression Analyses

Each outcome metric in every respondent group was evaluated individually. All variables were reduced to those associated with outcome by Elastic-Net Regularized Regression using the glmnet package in R v3.6.1. This feature set was further reduced by backwards stepwise feature selection, maximizing the Akaike Information Criterion (AIC). Statistical significance of each variable in the reduced feature set and odds ratio with respect to outcome were then assessed by multivariate Generalized Logistic Regression Model. Variables were also ranked by importance in a random forest model according to mean decrease in accuracy for each variable. Overall model predictive accuracy was quantified by Area-Under-the-ROC-Curve (AUC).

### Unsupervised Variable Clustering

For each group of survey respondents, supervised multivariate analyses of variables in association with each outcome were performed. Additionally, an unsupervised clustering of all survey response variables was completed in order to assess for “blocks” of highly correlated survey responses. Distance between variables was assessed by Spearman correlation, and clustering was performed by Partitioning Around Medioids (PAM), with optimal number of clusters selected by maximization of mean silhouette score. Pairwise statistical significance of association between variables was also assessed by Fisher’s Exact test. Separate heatmaps of variable-to-variable correlation were generated for each group of survey respondents across 1) all variables and 2) only the set of variables with pairwise p-values < 0.05, following Benjamini-Hochberg multiple-testing correction. This enabled direct visualization of highly correlated sets of survey responses in unbiased fashion.

## Results

### Medical students

There was a total of 179 medical student respondents to the survey. The overwhelming majority of medical student respondents were enrolled in dual-degree training programs (n = 165, 92%). There was a relatively balanced representation of trainees across all four clinical years of medical school (MS) (MS1, n = 69, 39%; MS2, n = 54, 30%; MS3, n = 18, 10%; MS4, n = 38, 21%) and between public (n = 77, 43%) and private institutions (n = 102, 57%). Demographics of survey respondents are listed in Table 1.

A total of 152 (85%) of MS respondents self-identified as being significantly stressed by the COVID-19 pandemic. Stressed respondents were more likely to be single or unmarried; however, they did not otherwise differ demographically or geographically from the non-stressed group. Stressed respondents were less likely to attend a public institution (n = 61, 40.1% vs n = 16, 61.5%, p = 0.042) and describe their medical training as being affected by the conversion to a virtual format for educational activities and advisor meetings (n = 115, 75.7% vs n = 14, 53.8%, p = 0.021). Time allocation during COVID-19 varied between stressed and non-stressed respondents, with the former describing less time spent on clinical duties (> 45% clinical time, n = 59, 38.8% vs n = 18, 69.2%, p = 0.004) and personal time (> 45% personal time, n = 10, 6.6% vs n = 5, 19.2%, p = 0.048). Stressed respondents described multiple ways in which their personal lives were adversely affected, including working from home (n = 89, 58.6% vs n = 7, 26.9%, p = 0.003), being physically isolated from friends/family due to work (n = 91, 59.9% vs n = 7, 26.9%, p = 0.002), and spending less time with partner (n = 54, 35.5% vs n = 15, 57.7%, p = 0.032) (Table 1 **and Supplemental Table 1**).

**Figure 1:**
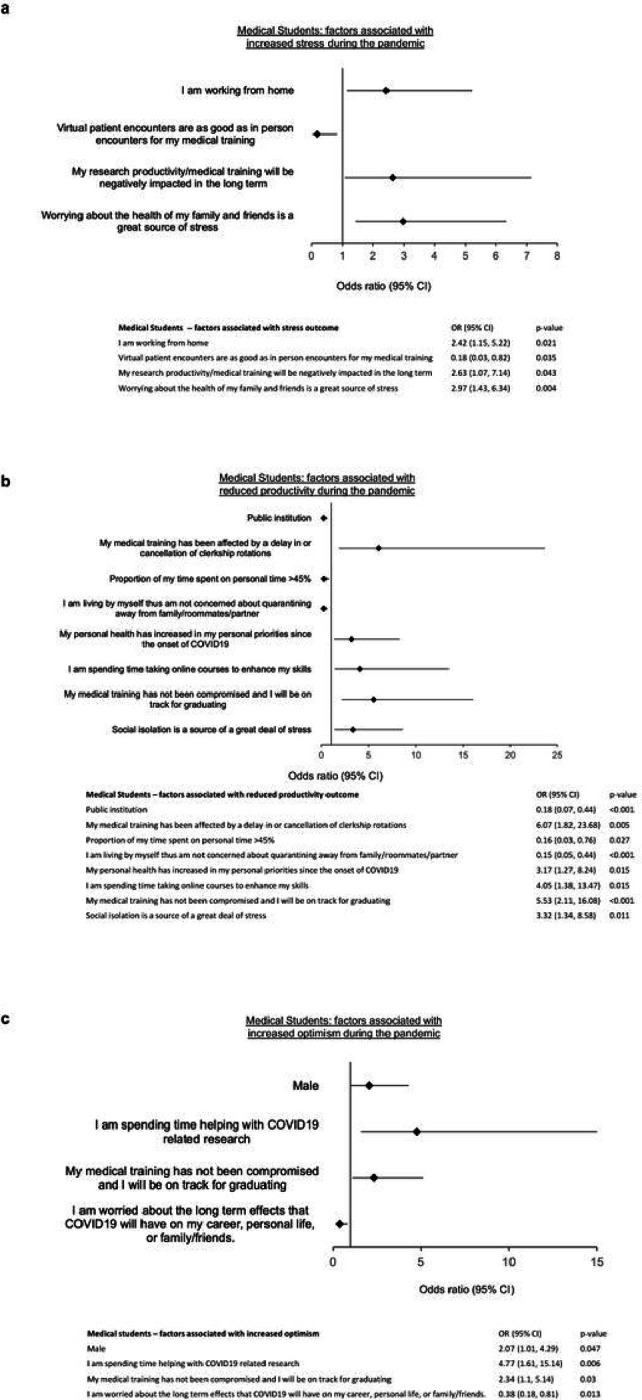
Medical student factors associated with increased a) stress, b) reduced productivity, c) increased optimism during the COVID-19 pandemic.

In a multivariate model for the outcome of stress, those who were working from home (OR 2.42, 1.15–5.22, p = 0.021), those who indicated their productivity will be negatively affected in the long term (OR 2.63, 1.07–7.14, p = 0.043) and those worrying about the health of family and friends (OR 2.97, 1.43–6.34, p = 0.004) were more likely to report increased stressed during the COVID-19 pandemic (Fig. 1a). The perception that virtual encounters are as good as in-person encounters for medical training was protective against stress among MS respondents (OR 0.18, 0.03–0.82, p = 0.035) (Fig. 1a).

Regarding productivity, a total of 117 (65%) MS respondents described their productivity as being adversely impacted by the pandemic (Table 2, **Supplemental Table 1**). A greater proportion of these trainees described working from home (n = 71, 60.7% vs n = 14, 35.9%, p = 0.007), being physically isolated from friends/family due to work (n = 71, 60.7% vs n = 16, 41%, p = 0.032), and being concerned with personal health (n = 90, 76.9% vs n = 21, 53.8%, p = 0.006) despite a 99% vaccination rate against COVID-19 in this cohort. Respondents with greater loss of productivity were also less likely to describe spending time preparing grant/fellowship applications (n = 4, 3.4% vs n = 5, 12.8%, p = 0.044) and working on collaborative projects (n = 21, 17.9% vs n = 13, 33.3%, p = 0.044). Consequently, this cohort expressed greater concerns regarding the negative impact of COVID-19 on the timeliness of graduation (n = 66, 56.4% vs n = 5, 12.8%, p < 0.001). Other concerns expressed in this cohort included personal health, social isolation, and financial consequence of the pandemic. Unsurprisingly, the overwhelming majority of trainees with impaired productivity self-identified as being significantly stressed (n = 108, 92.3% vs n = 27, 69.2%, p < 0.001) and with only a minority describing being optimistic about the future (n = 39, 33.3% vs n = 22, 56.4%, p = 0.019). (Table 2)

In a multivariate regression analysis for the outcome of impaired productivity, being at a public institution (OR 0.18, 0.07–0.44, p < 0.001), living by oneself (OR 0.15, 0.05–0.44, p < 0.001), having a high proportion of time (> 45%) spent on personal time (OR 0.16, 0.03–0.76, p = 0.027) were associated with a reduction in expressing impaired productivity. Having medical training delayed or clerkships canceled (OR 6.07, 1.82–23.68, p = 0.005), having personal health become a higher personal priority since the pandemic (OR 3.17, 1.27–8.24, p = 0.015), spending time taking online courses to enhance skills (OR 4.05, 1.38–13.47, p = 0.015), not having medical training compromised (OR 5.53, 2.11–16.08, p < 0.001) or saying social isolation is a source of stress (OR 3.32, 1.34–8.58, p = 0.011) were associated with impaired productivity (Fig. 1b).

In terms of optimism, female respondents were significantly less likely to report being optimistic than their male counterparts (n = 25, 37.9% vs n = 32, 64%, p = 0.005) (Table 3). Other than gender, the optimistic and non-optimistic cohorts did not differ by any other demographic or training characteristics. Respondents who were less optimistic were more likely to attribute feelings of stress, anxiety, and hopelessness due to the pandemic (n = 27, 40.9% vs n = 30, 60%, p = 0.042), describe depressive symptoms such as problems with sleep, fatigue, and changes in appetite (n = 25, 37.9% vs n = 30, 60%, p = 0.018), and report concern for the health of their friends and family (n = 34, 51.5% vs n = 37, 74%, p = 0.014) (Table 3). Of note, respondents who were less optimistic about the pandemic also described feeling stress due to many of the policy and political efforts in response to the pandemic, including concern regarding health disparities and gender inequity (n = 38, 57.6% vs n = 40, 80%, p = 0.011), and how the pandemic was managed at the local and national levels. Finally, there was greater concern regarding the potential long-term impact of COVID-19 on the careers and personal lives of those who were less optimistic about the pandemic (n = 22, 33.3% vs n = 33, 66%, p = < 0.001) See table 2 for characteristics of those who were more optimistic (Table 2).

By multivariate regression, those who expressed worry about the long-term effects of COVID-19 on career, personal life or on family/friends were less likely to be optimistic about the future (OR 0.38, 0.18–0.81, p = 0.013). Those who were male (OR 2.07, 1.01–4.29, p = 0.047), those who were spending time helping with COVID-19 related research (OR 4.77, 1.61–15.14, p = 0.006) and those indicating that their medical training has not been compromised (OR 2.34, 1.1–5.14, p = 0.03) were more likely to be optimistic about the future (Fig. 1c).

### Graduate students

A total of 319 respondents were in graduate school (GS). The majority of GS respondents were part of a dual-degree program (n = 305, 96%). Most respondents described working in a wet lab (n = 264, 82.8%). Fields of study represented included biological sciences (n = 279, 87.5%); computer and information science and engineering (n = 27, 8.5%); mathematical and physical sciences (n = 24, 7.5%); and social, behavioral, and economic sciences (n = 20, 6.3%). Forty-seven (14.7%) respondents were conducting COVID-19 related research. Remaining respondent characteristics are included in Table 1, Table 2, **Supplemental Table 1.**

Most respondents indicated that research/scholarly activities took up the majority of their time (n = 262, 82%) (**Supplemental Table 2**). However, in addition to research, GS respondents reported spending more time with friends/family (n = 214, 67.1%); on personal health (n = 197, 61.8%); with partners (n = 154, 48.3%); on exercise (n = 128, 40.1%); on hobbies (n = 116, 36.4%); on research (n = 77, 24.1%); on career success activities (n = 80, 25.1%) during the pandemic. The top five research activities were data analysis and experimental design (n = 284, 89%); reading scientific literature (n = 272, 85.3%); preparing manuscript drafts (n = 188, 58.6%); preparing grant, fellowship, or faculty applications (n = 151, 47.3%); preparing research seminars and/or posters for meetings or conferences (n = 118, 37%) (**Supplemental Table 1**).

The COVID-19 pandemic produced several academic and social stressors for this population. More than half of the respondents noted that they worry about the long-term effects that COVID-19 will have on their career, personal life, and/or family/friends (n = 172, 53.9%). One-third of respondents (n = 106, 33.2%) had their lab shut down, while 80.6% (n = 257) had experiments delayed or impaired and 11.6% (n = 37) were unable to perform any research activities. Important milestones were postponed: 41 (12.9%) respondents had their qualifying or thesis exam postponed and 45 (14.1%) had their transition back to medical school delayed. 74% of respondents (n = 236) agreed that their research productivity and/or medical training was being negatively impacted in the short-term, and 49.8% (n = 159) believed that it would affect their career in the long-term. Almost two-thirds of the respondents experienced a significant amount of stress, anxiety, hopelessness and/or depression (n = 201, 63%). This included 191 (54.5%) who reported sleep problems, decreased energy, changes in appetite, difficulty concentrating and/or restlessness (**Supplemental Table 1**).

**Figure 2:**
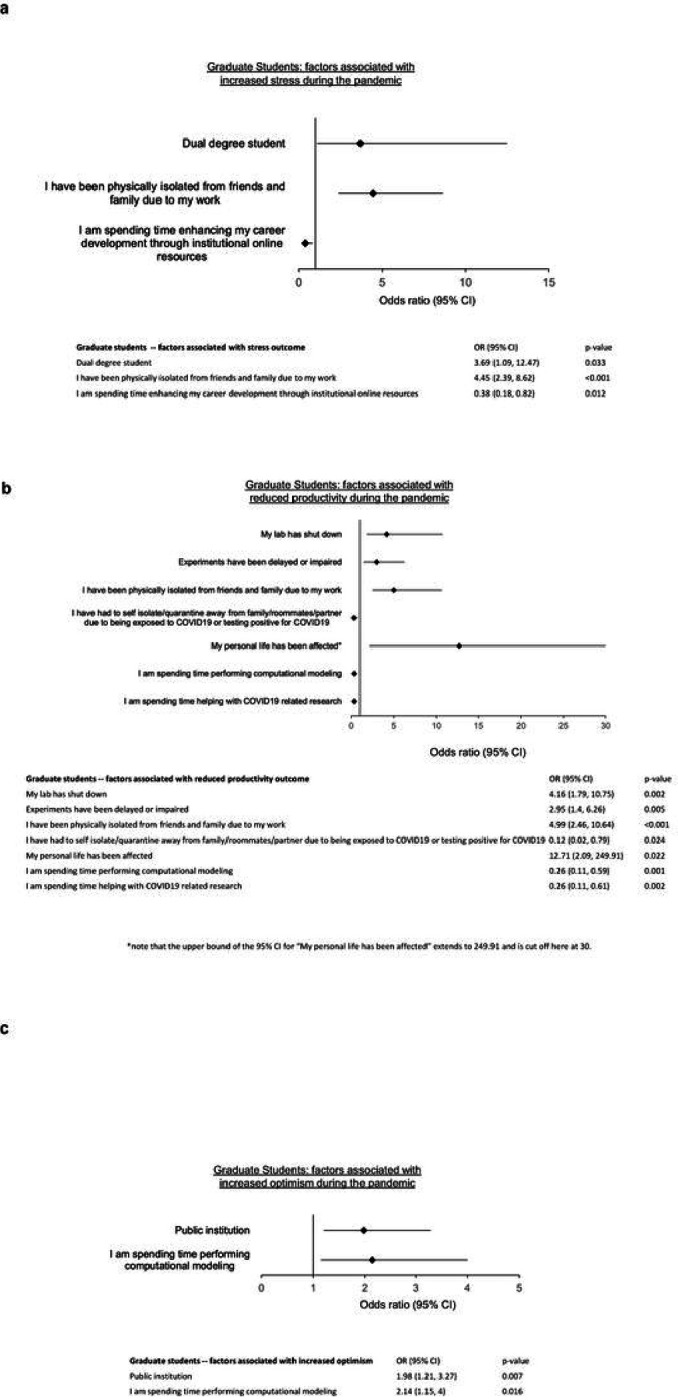
Graduate student factors associated with increased a) stress, b) reduced productivity, c) increased optimism during the COVID-19 pandemic.

In a multivariate regression analysis, those who indicated spending time enhancing career development through online resources were less likely to be stressed (OR 0.38, 0.18–0.82, p = 0.012). Those who were dual degree students (OR 3.69, 1.09–12.47, p = 0.033) or those who were physically isolated due to work (OR 4.45, 2.39–8.62, p = < 0.001) were more likely to be stressed (Fig. 2a).

In addition to affecting trainee stress levels, the COVID-19 pandemic had a significant and negative impact on GS training, productivity, and career development. The vast majority of students felt that virtual classrooms failed to adequately recapitulate in-person learning (83.5%, n = 248). While the majority of students reported that their labs did not shut down (65.0%, n = 193), most students did report that experiments were delayed due to the COVID-19 pandemic (82.2%, n = 244). Nearly all GS respondents felt that their research productivity would be impacted in the short term (79.5%, n = 236), and a significant proportion also felt that this impact would extend into the long-term (53.5%, n = 159) (**Supplemental Table 1**).

In a multivariate analysis, having to isolate from family, roommates, and/or partners due to being exposed to COVID-19 (OR 0.12, 0.02–0.79, p = 0.024), spending time performing computational modeling (OR 0.26, 0.11–0.59, p = 0.001) and spending time helping with COVID-19 related research (OR 0.26, 0.11–0.61, p = 0.002) were each associated with not having impaired productivity. Labs being shut down (OR 4.16, 1.79–10.75, p = 0.002), experiments being delayed/impaired (OR 2.95, 1.4–6.26, p = 0.005), physically isolating from friends/family due to work (OR 4.99, 2.46–10.64, p = < 0.001), and personal life being affected were associated with impaired productivity (Fig. 2b).

Interestingly, the COVID-19 pandemic had a significant effect on GS research topic of choice or intended career path for a subset of respondents. Thirty-one (9.7%) respondents shifted their research efforts to COVID-19 related topics, while twenty-six (8.2%) shifted their intended career path due to the pandemic. Expectedly, the overwhelming majority of students whose productivity was adversely effected reported as being stressed by the pandemic (90.4%, n = 224). Consistent with the stress and encumbered productivity among GS respondents, only 121 (55%) of students reported feeling optimistic (Table 3). In a multivariate analysis, being at a public institution (OR 1.98, 1.21–3.27, p = 0.007) and spending time performing computational modeling (OR 2.14, 1.15–4.0, p = 0.016) were associated with optimism for the future (Fig. 2c).

### Resident, Fellow, Junior Faculty (RFJF)

We received 178 responses from physician-scientists in post-graduate medical training and early career faculty positions, with an average age of 35.7 ± 5.7 years (range 19–51), 51% women, 9% Hispanic/Latino(a), and 3% Black/African American. Of note, the proportion of respondents for the latter two groups is similar to their proportion among MD-PhD graduates during this period.^4^ Among respondents, 74% were married or had a committed partner, and 48% had children. Completion of a formal dual degree training was reported in 60% of respondents, which is a significantly smaller proportion than represented amongst our medical and graduate student respondents (p < 0.001), while 23% were in a tenure track position (Table 1 **and Supplemental Table 1**).

**Figure 3:**
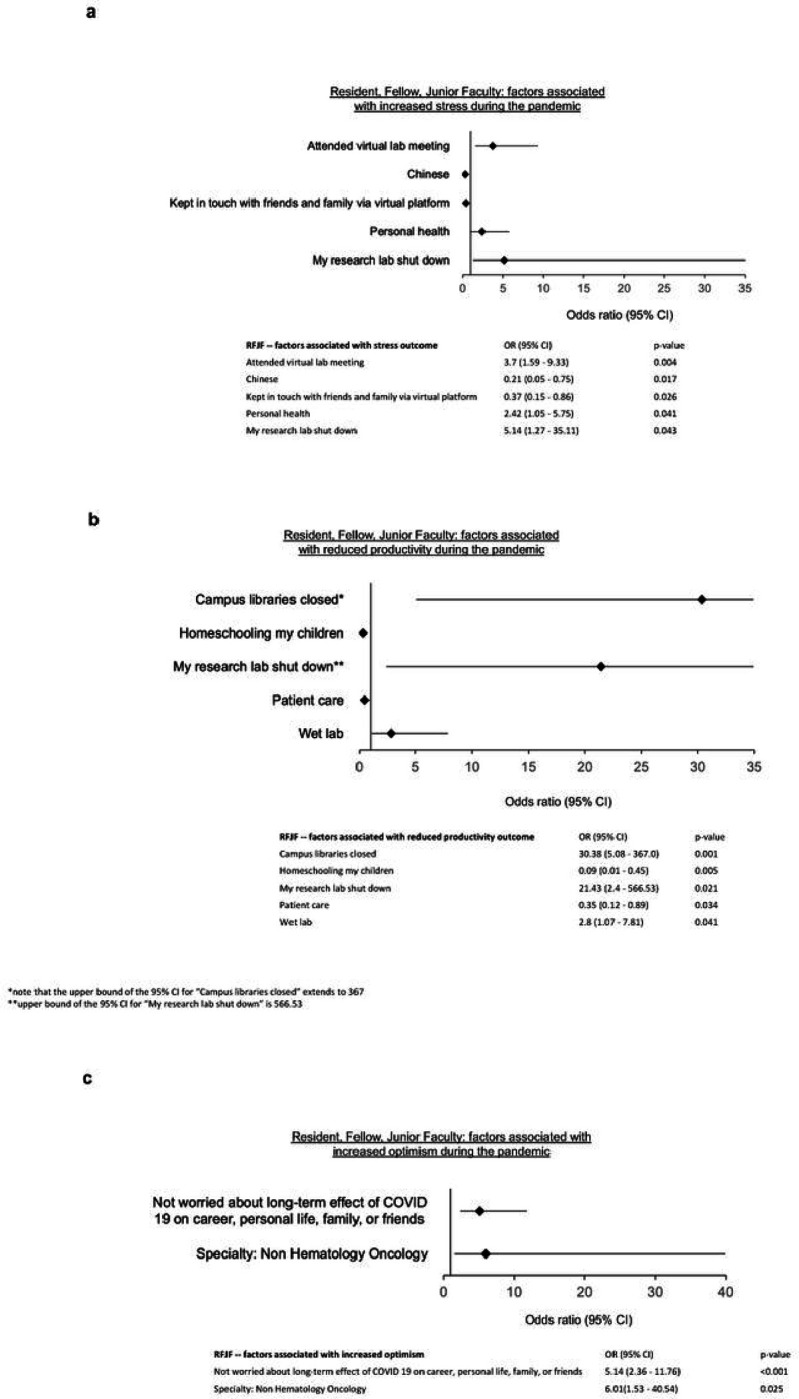
Resident, Fellow, and Junior Faculty factors associated with increased a) stress, b) reduced productivity, c) increased optimism during the COVID-19 pandemic.

Factors most predictive of stress during the pandemic, and which were reported by 85% of subjects, included research laboratory shutting down (OR 5.14, 1.27–35.11, p = 0.043), attending laboratory meetings virtually (OR 3.7, 1.59–9.33, p = 0.004), and worry about personal health (OR 2.42, 1.05–5.75, p = 0.041) (Fig. 3a). Protective factors included Chinese ethnicity (OR 0.21, 0.05–0.75, p = 0.017), and keeping in touch with friends and family via a virtual platform (OR 0.37, 0.15–0.86, p = 0.026). Collectively, these factors predict 88.8% of stress reported by RFJF during the COVID-19 pandemic.

Productivity impairment was described by 78% of RFJF respondents. A major contributor to decreased productivity included barriers to physical access to research facilities, including closure of campus libraries (OR 30.4, 5.08–367; p = 0.001), shutting down of research laboratories (OR 21.43, 2.4–567, p = 0.021), and working primarily in a wet laboratory setting (OR 2.8, 1.07–7.81, p = 0.041). Interestingly, homeschooling children (OR 0.09, 0.01–0.45, p = 0.005) and involvement in patient care (OR 0.35, 0.12–0.89, p = 0.034) were predictive of less reported impairment in productivity during the pandemic. Taken together, these factors predict 84.7% of the reported productivity impairment (Fig. 3b).

**Figure 4:**
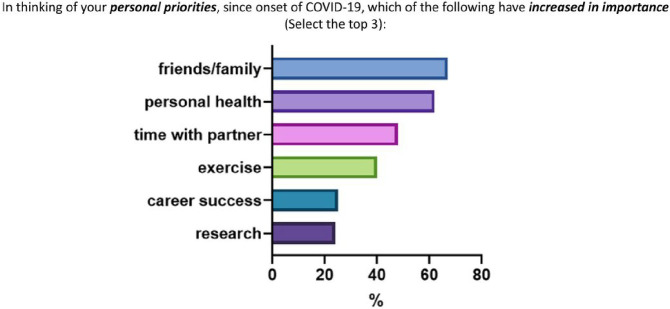
Personal priorities that have increased in importance during the pandemic in the total cohort.

RFJF respondents were much more likely to be optimistic about the future if they reported not being worried about the long-term effects of covid-19 on their career, personal life, or family and friends (OR 5.14, 2.36–11.76, p < 0.001), and if they were not specialized in hematology/oncology (OR 6.01, 1.53–40.54, p = 0.025) (Table 3, Fig. 3c). Finally, we found that respondents at every level of physician-scientist training and early career reported prioritization of friends/family (66%), personal health 62%, time with partner (48%) and exercise (40%), whereas career success was reported by only 25% and research by 24% of respondents (Fig. 4).

## Discussion

This national survey of physician-scientists and trainees provides key insights into the personal, psychosocial, educational, and professional implications of the COVID-19 pandemic in the United States. This work is a follow-up to a prior national survey conducted by our group and is meant to provide a cross-sectional point of reference of the major COVID-19 related stressors impacting the training and careers of physician-scientists.^3^ The data presented here reveal that the pandemic continues to have a significant effect on the stress, productivity, and perceived optimism of trainee and early career physician-scientists. Despite extensive prior work detailing these impacts, this survey suggests that many of the initial adverse consequences of the pandemic continue to have a persistent effect in this cohort. Taken together, the findings of the present study suggest that there is an ongoing need to address such concerns in a manner that is actionable and tailored to the specific training stage of early career physician-scientists and trainees.

### Medical students (MS)

Initial reports following the onset of COVID-19 described early adverse effects on MS respondents, including increased sense of social isolation, stress, and limited academic and scholarly productivity.^3,5,6^ While the negative impact of the COVID-19 pandemic was found to be consistent across all MS demographic cohorts, prior studies suggest that the effects were most severe amongst trainees who identified as female and URM.^3,6^ These trends raised concern that the differential impact of the pandemic amongst trainees may further exacerbate career disparities for groups traditionally underrepresented in medicine. Specifically, work by our group and others demonstrate that MS respondents expressed concern about the quality of their clinical education, given that abrupt transitions to virtual curricula and reduced clinical experience limited sub-specialty exposure and impacted residency selection.^3,7–10^

MS responses to this survey underscore the continued negative impact of COVID-19 on stress, productivity, and optimism. Multivariate regression modeling demonstrated that working from home, impaired productivity, and concerns regarding the health of friends/family were each independently associated with increased feelings of stress. Importantly, the only protective factor was the perception that virtual clinical encounters were adequate for supporting medical training. These responses suggest that while the factors associated with greater feelings of stress among medical students are diverse and span both personal and professional domains, limitations of hybrid or virtual education formats may provide an actionable area of improvement for mitigating stress in this cohort. These findings are again consistent with prior studies revealing medical student concern regarding the quality of their clinical training and exposure during COVID-19. ^3,7–10^ The results of this survey suggest that despite the necessity of virtual clinical curricula early in the pandemic, perceived limitations of virtual clinical education have a negative impact on the student learning experience. Reinstitution of in-person clinical and academic learning is likely to be beneficial for improving clinical confidence among trainees.

In addition to the direct personal and professional stressor caused by the pandemic, the indirect impact of the pandemic on productivity appears to be persistent among MS trainees. Unsurprisingly, those who described their clinical and academic progression as being significantly delayed reported greater levels of impaired productivity. While this survey did not evaluate the timing and extent of academic delays in this cohort, the finding that such trainees are experiencing delays is particularly relevant given the already increasing time to graduation among physician-scientist trainees. Interestingly, students attending public institutions were less likely to describe impaired productivity. It is not clear whether the protective effect of public-school attendance is due to selection bias among respondents or a reflection of the quality of the institutional responses to the pandemic, including better educational, academic, or psychosocial support resources. This is certainly an area that warrants additional investigation.

Finally, looking to the future of the pandemic, our results demonstrate a heterogenous response in perceived optimism among MS respondents. Optimistic respondents were more likely to be male, those who were able to engage in COVID-19 related research, and those who did not believe the timeline of their academic progression to be negatively impacted. The more optimistic perception of the future of the pandemic among males is consistent with our prior survey showing that the elevated stress early in the pandemic tended to be disproportionately impacting female MS.^3^ Prior studies have shown that the rates of self-reported depression were greater among female MS following the initial onset of the pandemic.^11^ It is likely that the reduced optimism described here is a sequela of this persistent depressive effect of the pandemic. This raises concern that the ongoing response to the pandemic has not been sufficient to support female trainees and demands greater understanding of the unique obstacles experienced by this cohort. Moreover, our survey results suggest that beyond female respondents, there continues to be a larger contingent of medical students who expressly state concern about the lasting impact of COVID-19 on their academic and professional careers, which warrants additional attention by academic and professional leadership.

### Graduate students (GS)

Prior studies on the GS experience during COVID-19 highlighted significant effects of the pandemic on mental health and academic productivity. Reported stressors early in the pandemic included isolation from friends and family, uncertainty about the future, and difficulty with social distancing.^11^ Similar to MS trainees, GS respondents also reported increased levels of disappointment, depression, anxiety, and suicidal ideation, with the greatest impact experienced among those historically underrepresented in medicine.^12–15^ Biomedical GS researchers in particular cited time management and decreased productivity as stressors during the COVID-19 pandemic, likely owing to the unique difficulties of conducting wet lab research during periods of mandated restriction to in-person activities.^12,16^ Multiple student commentaries during this time cite the obstacles in adapting new methods of maintaining research productivity in order to promote future career advancement.^17,18^

The results of the present survey clearly reveal that the COVID-19 pandemic continues to meaningfully affect GS trainees. Most GS respondents feel that their productivity—and in turn, their careers—have been delayed or impacted in both the near- and long-term. Despite a greater concern on productivity among students involved in non-computational research, negative perceptions regarding long-term productivity were shared across both wet and dry lab researchers. This is an impact that will necessarily warrant continued assessment for many years to come. Such gaps in trainee productivity and decreased optimism about the future will likely be negatively reflected in subsequent applications for training grants, career development awards, primary research funding, and post-doctoral or faculty applications.

Additionally, this survey confirms the pandemic intensified the stress felt by graduate trainees. Namely, our study emphasizes that the amount of stress the COVID-19 pandemic added to trainees’ lives is paramount to consider with careful intention. As medical and graduate trainees’ mental health and wellbeing are already known to be at risk—and often overlooked—the added stress of the pandemic has only exacerbated these issues.^19,20^ Indeed, over half of respondents indicated they experience physical symptoms of increased stress, depression, or anxiety greater than one year from the initial onset of the pandemic. With already established trends in worsening mental health among health care trainees and professionals, these findings indicate that continued attention must be paid to ensure that the well-being of physician-scientist trainees is supported.

### Resident, Fellow, Junior Faculty (RFJF)

In addition to high levels of stress and lost productivity, RFJF were more likely than MS and GS respondents to report financial difficulties early in the pandemic.^3^ Those with a dual degree reported more impairment in productivity than their non-dual degree holding counterparts.^3^ This appeared to be due, at least in part, to increased clinical duties resulting from increased demands on the healthcare system, as well as supply chain disruptions leading to limited access to essential research resources. In addition, women were more likely than their male counterparts to have increased home demands due to taking care of children, leading to further decreased productivity.

In the present survey, RFJF continued to report high levels of stress and loss of productivity because of the COVID-19 pandemic. These factors could potentially have devastating consequences for a generation of physician-scientists at a crucial point of their career. Indeed, many physicians abandon their research career at the stage of early career awards, notably at the transition from K to R awards.^21^ Stress and loss of productivity can also be compounded by the aforementioned worsening financial pressure. Interestingly, hematology/oncology respondents from this group were less optimistic about the future. While we did not identify a definite reason for this association, multiple factors including the need for frequent in-person treatment visits, lapses in cancer treatment and screening caused by the pandemic, and the immunocompromised nature of their patient population may be contributory.^22^ However, these findings may also be due to a selection bias in the subset of respondents sampled from this specialty. Irrespective of specialty-specific experiences, the findings from this survey indicate that personal and professional stressors related to the pandemic continue to have a protracted impact on RFJF. Such factors certainly add to the challenges experienced by this cohort as they attempt to establish their careers as independent investigators, and the consequences of these additive challenges need to be further studied.

## Figures and Tables

**Table 1.** Stress outcome

Medical students (n = 178)	Stressed (N = 152)	Not Stressed (N = 26)	P-value
Married or partnered	54 (35.5%)	11 (42.3%)	0.039
Public institution	61 (40.1%)	16 (61.5%)	0.042
Medical training affected virtual medical education or advisor meetings	115 (75.7%)	14 (53.8%)	0.021
Proportion of time spending on clinical duties increased	59 (38.8%)	18 (69.2%)	0.004
Proportion of time spending on personal time increased	10 (6.6%)	5 (19.2%)	0.048
Personal life affected by campus library closures	69 (45.4%)	4 (15.4%)	0.004
Personal life affected by working from home	89 (58.6%)	7 (26.9%)	0.003
I have been physically isolated from friends/family due to my work.	91 (59.9%)	7 (26.9%)	0.002
Personal life affected No effect	7 (4.6%)	7 (26.9%)	0.001
Increase in priority: time w/my partner	54 (35.5%)	15 (57.7%)	0.032
Spend time preparing drafts of manuscripts	51 (33.6%)	16 (61.5%)	0.006
Spend time preparing review articles	13 (8.6%)	6 (23.1%)	0.039
Spend time preparing figures or text for a collaborative manuscript	31 (20.4%)	10 (38.5%)	0.043
Virtual patient encounters are as good as in-person patient encounters for my medical training	5 (3.3%)	6 (23.1%)	0.002
My research productivity/medical training will be negatively impacted in the short-term	105 (69.1%)	6 (23.1%)	< 0.001
My research productivity/medical training will be negatively impacted in the long-term	46 (30.3%)	3 (11.5%)	0.048
My medical training has not been compromised and I will be on track for graduating	76 (50%)	23 (88.5%)	< 0.001
I am concerned about my medical training being compromised and not being prepared for internship/residency	70 (46.1%)	3 (11.5%)	< 0.001
The COVID-19 pandemic has caused me a significant amount of stress, anxiety, hopelessness and/or depression	92 (60.5%)	0 (0%)	< 0.001
The COVID-19 pandemic has caused sleep problems, decreased energy, changes in appetite, difficulty concentrating and/or restlessness	89 (58.6%)	0 (0%)	< 0.001
Uncertainty of not being able to finish my research or to graduate is a great source of stress	42 (27.6%)	0 (0%)	0.002
Worrying about my own health is a great source of stress	56 (36.8%)	0 (0%)	< 0.001
Worrying about the health of my family/friends is a great source of stress	102 (67.1%)	11 (42.3%)	0.015
I am worried about my own health from casual contact in the public	67 (44.1 %)	4 (15.4%)	0.006
Social isolation is a source of a great deal of stress	110 (72.4%)	0 (0%)	< 0.001
Financial consequences of the pandemic cause a great deal of stress	39 (25.7%)	0 (0%)	0.003
I am stressed out due to how the pandemic has been managed at the governmental level	117 (77%)	14 (53.8%)	0.013
I am worried about the long-term effects that COVID-19 will have on my career, personal life, and/or family/friends	81 (53.3%)	8 (30.8%)	0.034
Policy related stress	130 (85.5%)	18 (69.2%)	0.01
Impaired productivity outcome	108 (71.1%)	8 (30.8%)	< 0.001
Graduate students (n = 319)	Yes (N = 277)	No (N = 42)	P-value
Age (mean and standard deviation)	27.77 (23–42)	26.62 (21–30)	0.004
Experiments have been delayed or impaired.	230 (83%)	27 (64.3%)	0.004
Graduate training affected by campus library/computer center closures	104 (37.5%)	9 (21.4%)	0.042
Personal life affected by campus library closures	92 (33.2%)	5 (11.9%)	0.005
I have been physically isolated from friends/family due to my work.	174 (62.8%)	9 (21.4%)	< 0.001
Increase in priority: friends/family	193 (69.7%)	21 (50%)	0.011
Increase in priority: personal health	179 (64.6%)	18 (42.9%)	0.007
Increase in priority: No change	13 (4.7%)	8 (19%)	0.003
Spend time enhancing career development through institutional/online resources	36 (13%)	12 (28.6%)	0.009
Virtual classrooms recapitulate in-person learning	36 (13%)	14 (33.3%)	< 0.001
My research productivity/medical training will be negatively impacted in the short-term	213 (76.9%)	23 (54.8%)	0.002
My research productivity/medical training will be negatively impacted in the long-term	154 (55.6%)	5 (11.9%)	< 0.001
I have changed my intended career path/specialty intentions as a result of COVID-19	26 (9.4%)	0 (0%)	0.033
The COVID-19 pandemic has caused me a significant amount of stress, anxiety, hopelessness and/or depression	201 (72.6%)	0 (0%)	< 0.001
The COVID-19 pandemic has caused sleep problems, decreased energy, changes in appetite, difficulty concentrating and/or restlessness	191 (69%)	0 (0%)	< 0.001
Uncertainty of not being able to finish my research or to graduate is a great source of stress	174 (62.8%)	0 (0%)	< 0.001
Worrying about my own health is a great source of stress	125 (45.1%)	0 (0%)	< 0.001
Worrying about the health of my family/friends is a great source of stress	211 (76.2%)	16 (38.1%)	< 0.001
I am worried about my own health from casual contact in the public	133 (48%)	7 (16.7%)	< 0.001
Social isolation is a source of a great deal of stress	193 (69.7%)	0 (0%)	< 0.001
Financial consequences of the pandemic is a source of a great deal of stress	91 (32.9%)	0 (0%)	< 0.001
I am stressed out due to the political climate around health disparities and gender inequalities	179 (64.6%)	17 (40.5%)	0.003
I am stressed out due to how the pandemic has been managed at the governmental level	223 (80.5%)	17 (40.5%)	< 0.001
I am stressed out due to how the pandemic has been managed at the local level	170 (61.4%)	12 (28.6%)	< 0.001
I have been implementing stress-relieving practices, and I feel that I have a handle on my stress levels	121 (43.7%)	29 (69%)	0.002
I am worried about the long-term effects that COVID-19 will have on my career, personal life, and/or family/friends	163 (58.8%)	9 (21.4%)	< 0.001
I am optimistic about the future given the trajectory of the COVID-19 pandemic	96 (34.7%)	24 (57.1%)	0.005
Policy related stress	233 (84.1 %)	24 (57.1%)	< 0.001
Impaired productivity outcome	227 (82.2%)	24 (57.1%)	< 0.001
Optimism outcome	96 (35.2%)	24 (58.5%)	0.016
Resident, Fellow, Junior Faculty (n = 174)	Yes (N = 150)	No (N = 24)	P-value
Personal life affected by campus libraries closures	42 (28%)	2 (8.3%)	0.04
Personal life affected by working from home.	63 (42%)	5 (20.8%)	0.048
I have still been able to keep in touch with friends/family via virtual platforms.	94 (62.7%)	20 (83.3%)	0.048
I have to self-quarantine away from my family/roommates/partner due to being exposed to COVID-19 or because of COVID-19-related symptoms (without confirmatory COVID-19 testing).	23 (15.3%)	0 (0%)	0.047
Increase in priority: exercise	51 (34%)	3 (12.5%)	0.035
Spend time taking online courses to enhance skills	36 (24%)	1 (4.2%)	0.027
Virtual patient encounters are as good as in-person patient encounters for my medical training	15 (10%)	6 (25%)	0.047
My research productivity/medical training will be negatively impacted in the short-term (< 6months)	114 (76%)	10 (41.7%)	< 0.001
My research productivity/medical training will be negatively impacted in the long-term (> 6 months)	85 (56.7%)	5 (20.8%)	0.001
The COVID-19 pandemic has caused me a significant amount of stress, anxiety, hopelessness and/or depression	101 (67.3%)	0 (0%)	< 0.001
The COVID-19 pandemic has caused sleep problems, decreased energy, changes in appetite, difficulty concentrating and/or restlessness	78 (52%)	0 (0%)	< 0.001
Uncertainty of not being able to finish my research or to graduate is a great source of stress	76 (50.7%)	0 (0%)	< 0.001
Worrying about my own health is a great source of stress	68 (45.3%)	0 (0%)	< 0.001
Worrying about the health of my family/friends is a great source of stress	118 (78.7%)	6 (25%)	< 0.001
I am worried about my own health from direct patient contact of confirmed or suspected COVID-19 patients	67 (44.7%)	5 (20.8%)	0.028
I am worried about my own health from casual contact in the public	89 (59.3%)	3 (12.5%)	< 0.001
Social isolation is a source of a great deal of stress	99 (66%)	0 (0%)	< 0.001
Financial consequences of the pandemic is a source of a great deal of stress	46 (30.7%)	0 (0%)	0.002
I am stressed out due to the political climate around health disparities and gender inequalities	97 (64.7%)	10 (41.7%)	0.032
I am stressed out due to how the pandemic has been managed at the local level	92 (61.3%)	8 (33.3%)	0.01
I am worried about the long-term effects that COVID-19 will have on my career, personal life, and/or family/friends	100 (66.7%)	7 (29.2%)	< 0.001
Impaired Productivity Outcome	119 (82.1%)	11 (50%)	0.003

**Table 2.** Productivity outcome

Medical students (n = 156)	Impacted (N = 117)	Not Impacted (N = 39)	P-value
Age (mean and standard deviation)	26.24 (21–37)	25 (21–31)	0.028
Personal life affected by working from home	71 (60.7%)	14 (35.9%)	0.007
I have been physically isolated from friends/family due to my work.	71 (60.7%)	16 (41%)	0.032
I am living by myself thus am not concerned about quarantining from family members/roommates/partner	22 (18.8%)	16 (41%)	0.005
I have to self-quarantine away from my family/roommates/partner due to being exposed to COVID-19 or because of COVID-19-related symptoms (without confirmatory COVID-19 testing).	19 (16.2%)	1 (2.6%)	0.027
I have been exposed to COVID-19 or have symptoms (without confirmatory COVID-19 testing), but have no option to live in a different residence (hotel, institution facility).	15 (12.8%)	1 (2.6%)	0.075
Personal health has increased in priority	90 (76.9%)	21 (53.8%)	0.006
Spend time attending journal clubs by virtual platform	45 (38.5%)	22 (56.4%)	0.05
Spend time preparing grant/fellowship/faculty applications	4 (3.4%)	5 (12.8%)	0.044
Spend time preparing figures or text for a collaborative manuscript	21 (17.9%)	13 (33.3%)	0.044
Virtual patient encounters are as good as in-person patient encounters for my medical training	3 (2.6%)	6 (15.4%)	0.008
My research productivity/medical training will be negatively impacted in the short-term (< 6 months)	112 (95.7%)	0 (0%)	< 0.001
My research productivity/medical training will be negatively impacted in the long-term (> 6 months)	50 (42.7%)	0 (0%)	< 0.001
My medical training has not been compromised and I will be on track for graduating	51 (43.6%)	34 (87.2%)	< 0.001
I am concerned about my medical training being compromised and not being prepared for internship/residency	63 (53.8%)	5 (12.8%)	< 0.001
Uncertainty of not being able to finish my research or to graduate is a great source of stress	33 (28.2%)	4 (10.3%)	0.022
I am worried about my own health from direct patient contact of confirmed or suspected COVID-19 patients	35 (29.9%)	5 (12.8%)	0.034
Social isolation is a source of a great deal of stress	84 (71.8%)	17 (43.6%)	0.001
Financial consequences of the pandemic cause a great deal of stress	30 (25.6%)	3 (7.7%)	0.017
I am worried about the long-term effects that COVID-19 will have on my career, personal life, and/or family/friends	70 (59.8%)	14 (35.9%)	0.009
I am optimistic about the future given the trajectory of the COVID-19 pandemic	39 (33.3%)	22 (56.4%)	0.011
Stress outcome	108 (92.3%)	27 (69.2%)	< 0.001
Optimism outcome	39 (33.3%)	22 (56.4%)	0.019
Graduate students (n = 297)	Yes (N = 251)	No (N = 46)	P-value
Age (mean and standard deviation)	27.75 (21–42)	27.0 (23–32)	0.029
Field of research Computer and Information Science and Engineering (Computer and Network Systems, Information and Intelligent Systems)	17 (6.8%)	9 (19.6%)	0.009
My lab has shut down.	95 (37.8%)	9 (19.6%)	0.017
Experiments have been delayed or impaired.	217 (86.5%)	27 (58.7%)	< 0.001
I have been physically isolated from friends/family due to my work.	160 (63.7%)	12 (26.1%)	< 0.001
Spend time performing computational modeling	33 (13.1%)	14 (30.4%)	0.003
Spend time working collaboratively to outline an experimental plan for a study	49 (19.5%)	16 (34.8%)	0.021
Spend time preparing figures or text for a collaborative manuscript	84 (33.5%)	23 (50%)	0.032
Virtual classrooms recapitulate in-person learning	34 (13.5%)	15 (32.6%)	0.001
My research productivity/medical training will be negatively impacted in the short-term	236 (94%)	0 (0%)	< 0.001
My research productivity/medical training will be negatively impacted in the long-term	159 (63.3%)	0 (0%)	< 0.001
The COVID-19 pandemic has caused me a significant amount of stress, anxiety, hopelessness and/or depression	171 (68.1%)	19 (41.3%)	< 0.001
The COVID-19 pandemic has caused sleep problems, decreased energy, changes in appetite, difficulty concentrating and/or restlessness	169 (67.3%)	11 (23.9%)	< 0.001
Uncertainty of not being able to finish my research or to graduate is a great source of stress	151 (60.2%)	14 (30.4%)	< 0.001
Worrying about my own health is a great source of stress	106 (42.2%)	11 (23.9%)	0.019
Worrying about the health of my family/friends is a great source of stress	184 (73.3%)	25 (54.3%)	0.01
Social isolation is a source of a great deal of stress	160 (63.7%)	19 (41.3%)	0.004
Financial consequences of the pandemic is a source of a great deal of stress	76 (30.3%)	7 (15.2%)	0.036
I am stressed out due to how the pandemic has been managed at the governmental level	195 (77.7%)	27 (58.7%)	0.006
I am worried about the long-term effects that COVID-19 will have on my career, personal life, and/or family/friends	150 (59.8%)	13 (28.3%)	< 0.001
I am optimistic about the future given the trajectory of the COVID-19 pandemic	85 (33.9%)	27 (58.7%)	0.001
Stress outcome	227 (90.4%)	30 (65.2%)	< 0.001
Optimism outcome	85 (34.1%)	27 (61.4%)	0.003
Resident, Fellow, Junior Faculty (n = 157)	Yes (N = 131)	No (N = 26)	P-value
Dual degree	90 (68.7%)	11 (42.3%)	0.01
Type of laboratory: Wet Lab	87 (66.4%)	10 (38.5%)	0.007
Field of research: Biological Sciences (Environmental biology, Molecular Cellular Biosciences, Bioengineering)	102 (77.9%)	13 (50%)	0.003
My research lab has shut down.	35 (26.7%)	1 (3.8%)	0.011
Campus libraries have closed.	41 (31.3%)	1 (3.8%)	0.004
How spend time: Preparing research seminars and/or posters for meetings/conferences	34 (26%)	12 (46.2%)	0.039
How Spend time: Help with patient care	59 (45%)	18 (69.2%)	0.024
My research productivity/medical training will be negatively impacted in the short-term (< 6 months)	125 (95.4%)	0 (0%)	< 0.001
My research productivity/medical training will be negatively impacted in the long-term (> 6 months)	90 (68.7%)	0 (0%)	< 0.001
The COVID-19 pandemic has caused me a significant amount of stress, anxiety, hopelessness and/or depression	83 (63.4%)	10 (38.5%)	0.018
Uncertainty of not being able to finish my research or to graduate is a great source of stress	71 (54.2%)	3 (11.5%)	< 0.001
Worrying about my own health is a great source of stress	54 (41.2%)	5 (19.2%)	0.034
Worrying about the health of my family/friends is a great source of stress	98 (74.8%)	13 (50%)	0.011
I am stressed out due to how the pandemic has been managed at the governmental level	106 (80.9%)	15 (57.7%)	0.01
I have been implementing stress-relieving practices and I feel that I have a handle on my stress levels	53 (40.5%)	16 (61.5%)	0.048
Stress outcome	119 (90.8%)	18 (69.2%)	0.006
Policy related stress	117 (89.3%)	18 (69.2%)	0.008

**Table 3.** Optimism outcome

Medical students (n = 116)	Optimistic (N = 66)	Not optimistic (N = 50)	P-value
Gender: Female	25 (37.9%)	32 (64%)	0.005
My medical training has not been compromised and I will be on track for graduating	44 (66.7%)	23 (46%)	0.026
I have changed my research efforts to focus on COVID-19 related topics	9 (13.6%)	1 (2%)	0.042
I am concerned about my medical training being compromised and not being prepared for internship/residency	19 (28.8%)	25 (50%)	0.02
The COVID-19 pandemic has caused me a significant amount of stress, anxiety, hopelessness and/or depression	27 (40.9%)	30 (60%)	0.042
The COVID-19 pandemic has caused sleep problems, decreased energy, changes in appetite, difficulty concentrating and/or restlessness	25 (37.9%)	30 (60%)	0.018
Worrying about the health of my family/friends is a great source of stress	34 (51.5%)	37 (74%)	0.014
I am stressed out due to the political climate around health disparities and gender inequalities	38 (57.6%)	40 (80%)	0.011
I am stressed out due to how the pandemic has been managed at the governmental level	40 (60.6%)	42 (84%)	0.006
I am stressed out due to how the pandemic has been managed at the local level	30 (45.5%)	34 (68%)	0.016
I am worried about the long-term effects that COVID-19 will have on my career, personal life, and/or family/friends	22 (33.3%)	33 (66%)	< 0.001
Graduate students (n = 220)	Yes (N = 121)	No (N = 99)	
Gender: Female	51 (42.1%)	58 (58.6%)	0.015
Ethnicity: Hispanic	4 (3.3%)	11 (11.2%)	0.021
Region			
Midwest	33 (27.3%)	27 (27.3%)	0.003
Northeast	33 (27.3%)	48 (48.5%)	
South/Southeast	15 (12.4%)	8 (8.1%)	
Northwest/southwest	40 (33.1%)	16 (16.2%)	
Public Institution	72 (59.5%)	40 (40.4%)	0.005
Top 3 increase (onset) time w/my partner	51 (42.1%)	55 (55.6%)	0.048
My research productivity/medical training will be negatively impacted in the short-term	81 (66.9%)	82 (82.8%)	0.007
My research productivity/medical training will be negatively impacted in the long-term	47 (38.8%)	58 (58.6%)	0.004
The COVID-19 pandemic has caused me a significant amount of stress, anxiety, hopelessness and/or depression	61 (50.4%)	77 (77.8%)	< 0.001
The COVID-19 pandemic has caused sleep problems, decreased energy, changes in appetite, difficulty concentrating and/or restlessness	58 (47.9%)	72 (72.7%)	< 0.001
Uncertainty of not being able to finish my research or to graduate is a great source of stress	55 (45.5%)	67 (67.7%)	< 0.001
Worrying about my own health is a great source of stress	30 (24.8%)	51 (51.5%)	< 0.001
Worrying about the health of my family/friends is a great source of stress	76 (62.8%)	79 (79.8%)	0.006
I am worried about my own health from direct patient contact of confirmed or suspected COVID-19 patients	5 (4.1%)	17 (17.2%)	0.001
I am worried about my own health from casual contact in the public	36 (29.8%)	60 (60.6%)	< 0.001
Social isolation is a source of a great deal of stress	65 (53.7%)	71 (71.7%)	0.006
I am stressed out due to the political climate around health disparities and gender inequalities	61 (50.4%)	74 (74.7%)	< 0.001
I am stressed out due to how the pandemic has been managed at the governmental level	70 (57.9%)	89 (89.9%)	< 0.001
I am stressed out due to how the pandemic has been managed at the local level	49 (40.5%)	70 (70.7%)	< 0.001
I have been implementing stress-relieving practices, and I feel that I have a handle on my stress levels	64 (52.9%)	39 (39.4%)	0.046
I am worried about the long-term effects that COVID-19 will have on my career, personal life, and/or family/friends	47 (38.8%)	73 (73.7%)	< 0.001
Stress outcome	96 (80%)	91 (91.9%)	0.013
Policy related stress	80 (66.7%)	91 (91.9%)	< 0.001
Impaired productivity outcome	85 (70.8%)	86 (86.9%)	0.011
Resident, Fellow, Junior Faculty (n = 137)	Yes (N = 68)	No (N = 69)	
Increased proportion of time spent on personal time	63 (92.6%)	69 (100%)	0.028
I feel like my patients will suffer due to delayed presentation and/or disrupted in-person follow up	40 (58.8%)	56 (81.2%)	0.004
The COVID-19 pandemic has caused me a significant amount of stress, anxiety, hopelessness and/or depression	33 (48.5%)	45 (65.2%)	0.049
I am worried about my own health from casual contact in the public	30 (44.1%)	44 (63.8%)	0.021
I am stressed out due to how the pandemic has been managed at the governmental level	44 (64.7%)	61 (88.4%)	0.001
I am stressed out due to how the pandemic has been managed at the local level	33 (48.5%)	45 (65.2%)	0.049
I have been implementing stress-relieving practices and I feel that I have a handle on my stress levels	36 (52.9%)	24 (34.8%)	0.032
I am worried about the long-term effects that COVID-19 will have on my career, personal life, and/or family/friends	32 (47.1%)	53 (76.8%)	< 0.001
Policy related stress	51 (75%)	65 (94.2%)	0.005
